# Flavonoid constituents contribute to the low diastase activity of *Triadica cochinchinensis* honey through in vitro enzyme inhibition and in vivo transcriptional regulation

**DOI:** 10.1016/j.fochx.2026.104452

**Published:** 2026-09-12

**Authors:** Huizhi Jiang, Zhen Li, Tianyu Xu, Min Xu, Weixuan Chen, Shengyang He, Xiaobo Wu, Weiyu Yan, Qingfeng Zhang, Zhijiang Zeng

**Affiliations:** a Jiangxi Province Key Laboratory of Honeybee Biology and Beekeeping, Honeybee Research Institute, Jiangxi Agricultural University, Nanchang, Jiangxi 330045, China; b College of Life Science and Resources and Environment, Yichun University, Yichun, Jiangxi 336000, China; c Jiangxi Key Laboratory of Natural Product and Functional Food, College of Food Science and Engineering, Jiangxi Agricultural University, Nanchang 330045, China

**Keywords:** Honey quality, *α*-Amylase, (−)-Gallocatechin gallate, Naringenin chalcone, Hypopharyngeal gland

## Abstract

Diastase activity is a key international criterion for assessing honey quality, indicative of freshness and the absence of excessive heat treatment. This study found that the diastase activity of *Triadica cochinchinensis* honey (TCH) was only 3.45, significantly lower than other 11 common honeys with range of 9.96 to 23.06. Furthermore, its diastase activity abnormally decreased during honey ripening. Flavonoids have been reported to effectively inhibit diastase activity. The underlying mechanisms were investigated in vitro and in vivo based on the characteristic flavonoids in TCH identified by LC-MS/MS, i.e. unique (−)-gallocatechin gallate (GCG) and high-content naringenin chalcone (NGC). In vitro enzymatic assays indicated that GCG and NGC inhibited *α*-amylase activity via mixed-type. The binding of GCG and NGC on *α*-amylase was further characterized by fluorescence quenching and molecular docking. In vivo studies revealed that dietary supplementation of GCG and NGC suppressed the *α-amylase* gene expression in the hypopharyngeal glands of honey-ripening bees (15-day-old) by 78.92% and 67.71%, respectively, and in foragers (25-day-old) by 91.14% and 56.48%, respectively. Network pharmacology analysis linked this suppression by interfering with four α-amylase-related pathways.

## Highlights

• *Triadica cochinchinensis* honey (TCH) exhibits markedly lower diastase activity than 11 other honeys.
• (−)-Gallocatechin gallate (GCG) and naringenin chalcone (NGC) in TCH exerted a direct inhibitory effect on α-amylase activity.
• Dietary supplementation of GCG and NGC downregulated the *α-amylase* gene expression in the hypopharyngeal glands of worker bees *(Apis mellifera*).
• Diastase activity, a commonly used quality evaluation indicator of honey, may be unsuitable for TCH.

## Graphical abstract

**Figure:**
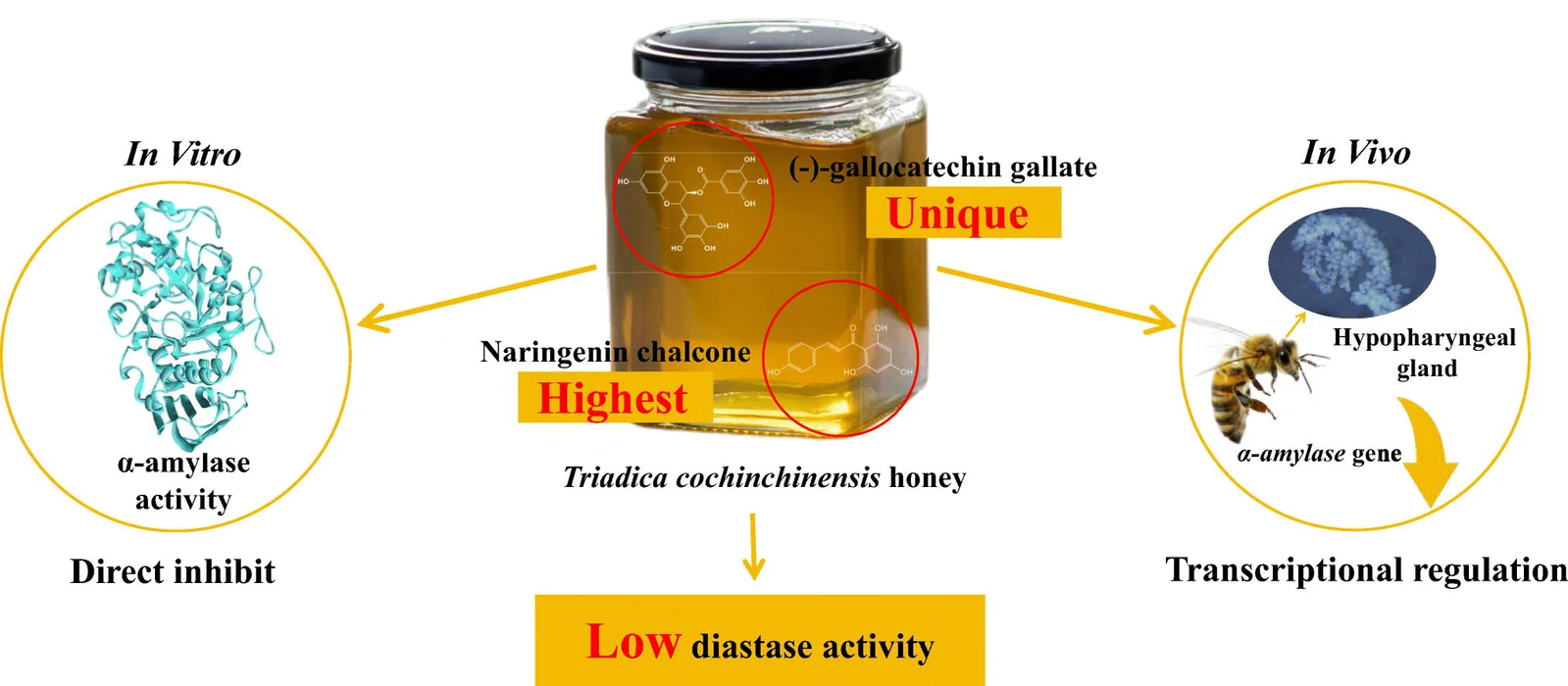

## 1 Introduction

Diastase, the starch-digesting enzymes in honey, mainly consists of α-amylase, accompanied by β-amylase (Yiğit et al., 2024). α-amylase hydrolyzes starch chains at random locations, producing a variety of dextrins, while β-amylase produces maltose from the non-reducing end of the starch or dextrin chain (Sak-Bosnar & Sakač, 2012). The hydrolysis processes enhance the utilization efficiency of carbohydrates and provide energy for honey bees (Zhang et al., 2025). More than 80% diastase in honey originate from the hypopharyngeal glands of worker bees, while a small amount may be derived from plant sources (Babacan & Rand, 2007). During the conversion of nectar into honey, worker bees secrete enzymes such as diastase and sucrase (invertase) to break down starch or disaccharides present in nectar into monosaccharides (Wang et al., 2022).

Honey is widely popular as a natural sweetener in the world (Pourmoradian et al., 2025). Diastase activity is widely used as a critical quality parameter, directly indicating the freshness and processing suitability of honey (Pasias et al., 2017; Takahashi et al. 2025). Diastase activity is defined as the amount of enzyme that converts 0.01 g of starch to dextrins in one hour at 40 °C and is expressed in Schade units or diastase number (Bell & Grainger, 2023). In accordance with international standards set by Codex Alimentarius Commission and European Honey Directive 2001/110/EC, honey diastase activity must be greater than 8 for trade and export (Sęk et al., 2023). Same requirements also included in national standards of Canada (Thrasyvoulou et al., 2018), Saudi Arabia (Raweh et al., 2023) and China (NY/T 4644-2025) (Ministry of Agriculture and Rural Affairs of the People's Republic of China, 2025). However, some special honeys exhibit naturally low diastase activity, which challenges the universal applicability of this index.

*T. cochinchinensis* honey (TCH) is a representative honey with naturally low diastase activity. *T. cochinchinensis*, a deciduous tree or shrub, is widely distributed across East to Southeast Asia, including regions of southern China, India, Myanmar, Laos, and Indonesia (Cai et al., 2024; Wang et al., 2016). It is a major monofloral nectar source in subtropical summers (from June to July) and characterized by a long flowering period and high nectar secretion, which can support the production of TCH and bring substantial profit for beekeepers (Jiang et al., 2025). TCH has attracted growing interest due to its unique flavor profile and medicinal values (Yi et al., 2024). However, TCH exhibits naturally lower diastase activity compared to common commercial varieties (Luo et al., 2021), and this difference may not be attributable to heating, processing, or storage conditions.

As commonly and naturally occurring secondary metabolites in honey, flavonoids have been reported to be effective in inhibiting *α*-amylase activity (Moore et al., 2025; Yang et al., 2021). For example, Xiao et al. (2025) reported that anthocyanins and their aglycones can inhibit α-amylase, and hydroxyl groups in the B-ring strengthen the inhibitory activity. Wang et al. (2025) also found that three flavonoids featuring 5,6-dihydroxyflavone structures showed a competitive mixed inhibition against α-amylase. TCH is rich in flavonoid components, including naringenin, rutin, quercetin, and so on (Yu et al., 2023). Yet few studies have explored the relationship between TCH-derived flavonoids and low diastase activity of TCH.

This study systematically investigated the phenomenon and mechanisms of low diastase activity in TCH. It first compared diastase activity in TCH with that of 11 other honeys and further tracked changes during TCH ripening. Then, LC-MS/MS was used to identify characteristic flavonoids in TCH. Two hypotheses were tested herein: first, that characteristic flavonoids present in TCH directly inhibit α-amylase activity; and second, that consumption of these flavonoids by worker bees suppresses α-amylase secretion in their hypopharyngeal glands. These hypotheses were examined through in vitro enzymatic assays and honey bee feeding experiments. Finally, network pharmacology was used to further explore the relationships among the flavonoids, potential targets, and α-amylase-associated pathways.

## 2 Materials and methods

### 2.1 Samples collection

TCH and 11 other common types of Chinese commercial honey were chosen for this experiment*.* TCH and *Brassica campestris* honey (BCH) were collected from the Weimin apiary (*Apis mellifera*) in Yongxiu County, Jiangxi Province, China (29°01 N, 115°29 E) in 2025. Nine other monofloral honeys, including *Vitex negundo* L. *var.* honey (VNH), *Robinia pseudoacacia* honey (RPH), *Ziziphus jujuba* honey (ZJH), *Tilia tuan* honey (TTH), *Eriobotrya japonica* honey (EJH), *Litchi chinensis* Sonn honey (LSH), *Citrus reticulata* honey (CRH), *Lycium chinense* Miller honey (LMH), *Camellia oleifera* honey (COH) and one polyfloral honey (POH) were collected from local *Apis mellifera* apiaries in different places across China. All the above-mentioned samples were capped honey, which is naturally mature as the nectar has been thoroughly processed by worker bees and sealed in honeycomb cells for over 15 days.

Freshly deposited honey refers to nectar collected by worker bees and stored in honeycomb cells for no more than one day. The freshly deposited honey of *T. cochinchinensis* (TCFDH) and *B. campestris* (BCFDH) were collected during their flowering periods from March to July 2025. Empty honeycombs were placed in the experimental colonies (*Apis mellifera*) at 5 a.m. Then, the honey samples in the honeycombs were collected at 5 p.m. *T. cochinchinensis* nectar (TCN) was collected from the nectary of *T. cochinchinensis* plant using a micro aspirator (Beijing Dalong, Beijing, China).

Microscopic melissopalynological analysis was first conducted to identify the botanical origin of 11 types of monofloral honeys. Sample pretreatment procedure was performed following Song et al. (2012). A light microscope equipped with a camera (DS-Fi3, Nikon Corporation, Tokyo, Japan) was used to observe pollen morphology and count the native pollen rate. The equatorial and polar pollen morphology of 11 of monofloral honeys was characterized in Fig.S1, and all exhibited high native pollen rate (Table S1), indicating that they meet the monofloral honey requirement (> 45%) (Bratosin et al., 2025).

### 2.2 Chemicals and reagents

Sodium acetate, acetic acid, iodine, potassium iodide, sodium chloride, and starch were sourced from Sinopharm Chemical Reagent Co., Ltd. (Shanghai, China). Acetonitrile, formic acid and methanol were of LC-MS-grade and obtained from Merck (Darmstadt, Germany). GCG, NGC and other remaining standards (purity ≥98%) were purchased from Bidepharm Co., Ltd. (Shanghai, China). Sucrose, sodium hydroxide and ethanol were obtained from Xilong Scientific Co., Ltd. (Shantou, China). Porcine pancreatic α-amylase (PPA), potassium dihydrogen phosphate, potassium dihydrogen phosphate trihydrate and corn starch were bought from Yuanye Bio-Technology Co., Ltd. (Shanghai, China). Acarbose was sourced from Macklin Biochemical Co., Ltd. (Shanghai, China).

### 2.3 Determination of diastase activity in honey samples

The diastase activity of honey samples was determined in accordance with the Chinese National Standard (GB/T18932.16-2003) (General Administration of Quality Supervision, Inspection and Quarantine of the People's Republic of China, 2003). Briefly, 5.0 g of honey sample was dissolved in 15 mL of water. Subsequently, 2.5 mL of acetate buffer (pH 5.3) and 1.5 mL of 0.5 mol/L sodium chloride were added, and the solution was finally diluted to 25 mL with water. An aliquot of 10 mL of prepared honey solution was mixed with 5 mL of starch solution (0.2 g/L). Starting from the 5th minute, 1.0 mL of the mixture was taken out every 15 min. After adding 10.0 mL of iodine solution, the absorbance at 660 nm was measured using a spectrophotometer (UV752N, Yoke Instrument Co., Ltd., Shantou, China). This process was repeated until the recorded absorbance fell below 0.235. A calibration curve plotting absorbance versus time was constructed to determine the time at which the absorbance of the sample mixture reached 0.235. Each experiment was repeated with five biological replicates.

### 2.4 LC-MS/MS analysis

To conduct quantitative and qualitative analysis of flavonoid constituents in TCH, TCN and 11 other types of honey samples were first lyophilized and powdered, respectively. Then, 0.20 g of honey sample was mixed with 5 mL of 70% (v/v) methanol and 100 μL of the internal standard working solution containing daidzein, rutin and (−)-gallocatechin (each at 4000 nmol/L). The mixture was subjected to 30 min of ultrasonication, followed by centrifugation (12,000 r/min, 4 °C, 5 min). The obtained supernatant was filtered (0.22 μm) into a glass vial, then analyzed by an ultra-performance liquid chromatography system (ExionLC™ AD, SCIEX, Framingham, MA, USA) coupled with tandem mass spectrometry (QTRAP®6500+, SCIEX, Framingham, MA, USA). The chromatographic condition, mass spectrometry condition, flavonoid standard curves construction and linearity range determination, and qualitative and quantitative parameters criteria were performed as described in our previous study (Jiang et al., 2024). Each experiment was repeated with three biological replicates.

### 2.5 Assessment of α-amylase inhibition by GCG and NGC

#### 2.5.1 Determination of α-amylase inhibitory activity

The inhibitory activity of α-amylase was determined according to the method of Ho and McKay (1999) with some modifications. Briefly, α-amylase was dissolved in phosphate buffer (PB, 20 mM, pH 6.9) at a concentration of 1 U/mL. The substrate, corn starch, was dissolved in boiling PB at a concentration of 1% (m/v). GCG and NGC were dissolved in 75% ethanol with different concentrations (5–30 mg/mL). These solutions were pre-incubated in a water-bath at 37 °C. In a 0.5 mL centrifuge tube, 50 μL of α-amylase solution, 10 μL of GCG (or NGC) and 100 μL of substrate were mixed. The mixture was incubated at 37 °C for 15 min. Then, 100 μL of reactant was mixed with 200 μL of DNS reagent, and placed in boiling water for 5 min. After cooling, the absorbance at 540 nm was measured using a 96-well plate reader (M2 microplate reader, Molecular Device Co., Ltd., CA, USA). The inhibitory activity of α-amylase by GCG and NGC was calculated by the following equation, with acarbose as the positive control:

(1) $$\mathrm{inhibition}\;\left(\%\right)=\frac{\left(A_{1}-A_{2}\right)-\left(A_{3}-A_{4}\right)}{\left(A_{1}-A_{2}\right)}\times 100$$

where A1 and A2 were the absorbance of negative control and its blank (without enzyme), while A3 and A4 were the absorbance of reactant with inhibitor adding and its corresponding blank, respectively. Each experiment was repeated with three biological replicates.

#### 2.5.2 Inhibition kinetics analysis

The activity of α-amylase was measured at different substrate concentrations (0.1–1% (m/v)) with fixed concentrations of GCG and NGC (0 or 0.625 mg/mL). The specific measurement steps are the same as those in 2.5.1. The inhibition type was determined through the Lineweaver-Burk plot of the Michaelis-Menten equation.

(2) $$\frac{1}{V}=\frac{K_{m}}{V_{\max}}\frac{1}{\left[S\right]}+\frac{1}{V_{\max}}$$

where V was the enzyme velocity, Vmax was the maximum enzyme velocity, Km represented the Michaelis constant, and [S] represented the substrate concentration. Each experiment was repeated with three biological replicates.

### 2.6 Characterization of the binding of GCG and NGC to α-amylase

#### 2.6.1 Fluorescence quenching analysis

An aliquot of 4.9 mL α-amylase (0.6 U/mL) solution was mixed with 100 μL of inhibitor with different concentration. After standing at room temperature for 5 min, the fluorescence spectrum of the mixture was recorded between 300 and 500 nm with excitation at a wavelength of 280 nm (970CRT fluorescence spectrophotometer, Shanghai Scientific Instruments Co., Ltd., Shanghai, China). Each experiment was repeated with three biological replicates.

#### 2.6.2 Molecular docking

Molecular docking was performed using AutoDock Vina program (http://vina.scripps.edu/). The 3D molecular structures of GCG and NGC were generated by Chem3D 19.0 software (CambridgeSoft Corporation, USA), and the 3D structure of amylase (PDB ID: 1HX0) was downloaded from RCSB Protein Data Bank (http://www.rcsb.org/pdb/home.do). AutoDock Tools were used for adding hydrogen atoms, distributing Gasteiger particle charges, and merging nonpolar hydrogen atoms. According to the structure features of 1HX0, the center coordinates of docking pocket are (37.2, 18.9, 60.4), and its size is (25.8, 21.1, 26.3 Å). Docking was performed using the Vina 1.2.5 software. Ten conformations after docking were obtained, and they were sorted from low to high based on the binding free energy.

### 2.7 Analysis of α-amylase gene relative expression in hypopharyngeal glands

#### 2.7.1 Feeding treatments and hypopharyngeal gland collection

Honey bees (*Apis mellifera*) were maintained at the Honeybee Research Institute of Jiangxi Agricultural University, China (28.46° N, 115.49° E). A frame of sealed brood nearing emergence was removed from the honey bee colonies and placed overnight in a climate-controlled chamber (temperature: 34 °C; relative humidity: 65%). The newly born worker bees were transferred to a plastic basin with inner walls coated with cooking oil to prevent escape. 700–800 worker bees were marked on their thoraxes using colored dye. After being lightly sprayed with a sugar solution, the marked worker bees were introduced into the experimental colony.

On day 12, marked worker bees (now 12-day-old) were collected from the colony and housed in six plastic cups, with 20–30 worker bees each. The groups were assigned as follows: Cup 1 (Control group 1) received a 50% sucrose solution; Cup 2 (Treatment group 1) received a 50% solution of TCH; Cup 3 (Treatment group 2) received a 50% solution of BCH; Cup 4 (Control group 2) received a 50% sucrose solution with 0.3% ethanol; Cup 5 (Treatment group 3) received a 50% sucrose solution supplemented with 57.58 ng/g GCG and 0.3% ethanol; Cup 6 (Treatment group 4) received a 50% sucrose solution supplemented with 872.23 ng/g NGC and 0.3% ethanol. The concentrations of GCG and NGC were consistent with their concentrations in TCH. After feeding 3 days, the hypopharyngeal glands of honey bees (15-day-old) were dissected. Worker bees were anesthetized with carbon dioxide to separate their heads. The heads were fixed on a wax dissection plate with the front side up, and dissections were performed under a stereomicroscope (×20). Sterile blades were used to incise the head capsule along the inner side of the eyes to expose the internal tissues. The hypopharyngeal glands, which are located on the top of brain on both sides and exhibit typical string of pearls appearance, were carefully clamped and separated using fine forceps. The isolated pair of hypopharyngeal glands was immediately frozen in liquid nitrogen and stored at −80 °C for subsequent analysis of *α-amylase* gene relative expression.

Using the same methodology, marked honey bees were collected again on day 22 (22-day-old) and subjected to an identical feeding experiment with the six treatment groups.

#### 2.7.2 Quantitative real-time PCR (qRT-PCR)

Total RNA was extracted from the sampled hypopharyngeal glands with the Trans-Zol Up RNA Kit (TransGen Biotech, Beijing, China). RNA was used to synthesize cDNA using a reverse transcription kit (TaKaRa, Tokyo, Japan). The housekeeping gene *gapdh* of *Apis mellifera* was used as an internal reference gene. Quantitative real-time PCR primers were designed using the mRNA sequences of the *α-amylase* gene in Primer Premier 5.0. The primer sequences are listed in Table S2.

qRT-PCR reaction system (10 μL) consisted of 1 μL of cDNA, 5 μL of TB Green, 3 μL of ddH_2_O, 0.4 μL each of forward and reverse primers, and 0.2 μL Rox. Amplification conditions of the qRT-PCR were as follows: 50 °C for 2 min, pre-denaturation at 95 °C for 10 min, followed by 40 cycles of 95 °C for 10 s, (*α-amylase* at 58 °C and *gapdh* at 52.9 °C) for 1 min. For each sample, the specificity of the PCR amplification was verified by melting curve analysis. There were 8 biological replicates per group (a pair of hypopharyngeal glands of a honey bee was a biological replicate), and each reaction had 4 technical replicates for each gene. Expression levels were calculated using the 2^−(∆∆Ct)^ method (Livak & Schmittgen, 2001).

### 2.8 Network pharmacology analysis

Firstly, the SMILES constructions of GCG and NGC were obtained from PubChem database (https://pubchem.ncbi.nlm.nih.gov, accessed on 7 January 2026) and uploaded to the SwissTargetPrediction tool (https://www.swisstargetprediction.ch/, accessed on 7 January 2026) to obtain the compound-related targets. Then with “α-amylase inhibition” as the keyword, the related targets were collected using the Genecards (https://www.genecards.org/, accessed on 7 January 2026). The common targets between GCG and NGC and α-amylase inhibition were submitted to the database of Metascape (https://metascape.org, accessed on 7 January 2026) for KEGG enrichment analysis.

### 2.9 Statistical analysis

Data were expressed as mean ± standard deviation (SD). Data normality was confirmed via Shapiro–Wilk tests. Comparisons between two groups were conducted using the unpaired *t*-test, while comparisons among multiple groups were performed using one-way analysis of variance (ANOVA) followed by Tukey's honestly significant difference (HSD) test for post hoc multiple comparisons. Statistical analysis was performed using SPSS software (version 27, IBM Corp., NY, USA). Differences were considered statistically significant when *P* < 0.05. Graphical representations of experimental findings were generated using Origin 8.5 (Northampton, MA, USA) and GraphPad Prism 9.5 (San Diego, CA, USA).

The mass spectrometry data acquisition was collected via Analyst 1.6.3 software (AB SCIEX, MA, Framingham, USA) and subsequently processed in Multiquant 3.0.3 software (AB SCIEX, Framingham, MA, USA). Metabolite quantification was validated by comparing the retention times and peak shape information of the corresponding standards, followed by integral correction of the mass spectrometry peaks.

## 3 Results and discussion

### 3.1 Diastase activity analysis of different kinds of honey

**Fig. 1:**
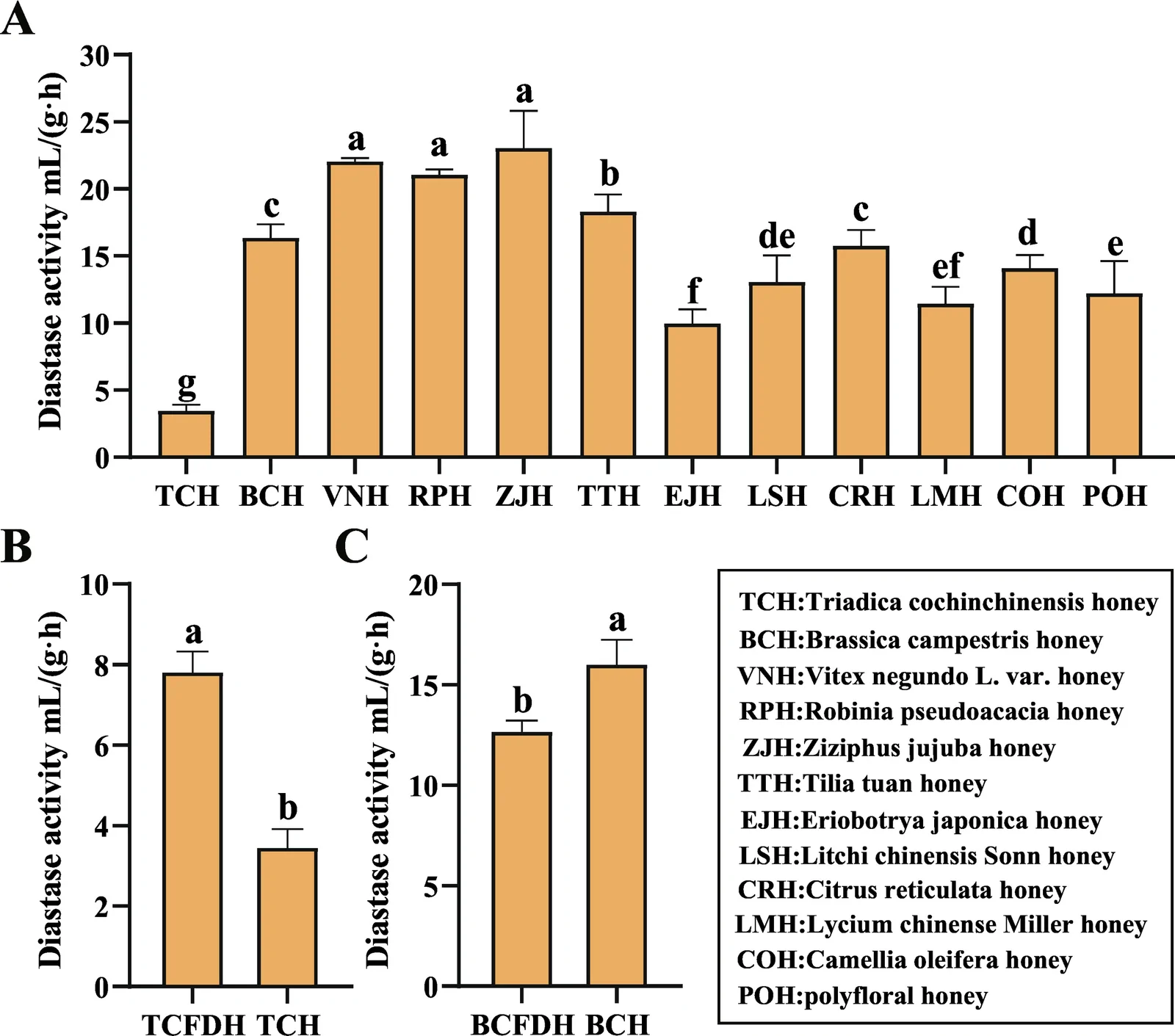
Diastase activity of honey. (A) The diastase activity of *Triadica cochinchinensis* honey (TCH) and 11 other common commercial honeys. (B) The diastase activity of *T. cochinchinensis* freshly deposited honey (TCFDH) vs TCH. **(**C) The diastase activity of *Brassica campestris* freshly deposited honey (BCFDH) vs *B. campestris* honey (BCH). Data in (A) were analyzed using one-way analysis of variance (ANOVA), followed by Tukey's honestly significant difference (HSD) post hoc test, while data in (B) and (C) were analyzed using an unpaired *t*-test. Different lowercase letters indicated significant differences (*P* < 0.05).

The diastase activity of TCH and 11 other common commercial honeys in China was compared. As shown in Fig. 1A, distinct variations in diastase activity were observed among different varieties of honey. Except for TCH, diastase activity of the other 11 honeys ranged from 9.96 to 23.06. In contrast, the diastase activity of TCH was only 3.45, which was significantly lower than that of the other 11 honeys (*P* < 0.05). This is consistent with research by Zhang et al. . (2025) and Jiang et al. . (2025), who both reported that TCH has naturally low diastase activity. The international standard requires that the diastase activity in honey should not be less than 8 (Ren et al., 2022). Hence, among the 12 commercial honeys, only TCH did not meet the requirement.

Generally, diastase in honey has two sources: nectar origin and secretion by worker bees during honey ripening (Erban et al., 2021; Lupu et al., 2025). Hence, the diastase activities in TCH and BCH before and after honey bee brewing were determined and compared. As shown in Fig. 1B, the diastase activity of TCFDH (7.81) was markedly higher than that of TCH (3.45). The results suggested that the low diastase activity of TCH was not due to its nectar origin but mainly resulted from the ripening process. This observation aligned with the finding by Liu et al. (2023), which indicated that the diastase activity of TCH decreases with increasing maturity. Generally, diastase activity in honey tends to increase during maturation due to water reduction and worker bee brewing (Balasubramanyam, 2020; Zhang et al., 2021). This trend was confirmed during BCH maturation in this study. As shown in Fig. 1C, BCH showed significantly higher diastase activity than BCFDH after ripening by worker bees for over 15 days. Wu's (2022) study also reported that the diastase activity of BCH increases with maturation.

In contrast to the increase in diastase activity in BCH after maturation, TCH showed the opposite trend. This phenomenon echoed the accelerated loss of diastase activity in mānuka honey, which is driven by its unique chemical composition, particularly methylglyoxal and 3-phenyllactic acid (Bell & Grainger, 2023). Accordingly, we hypothesized that during the maturation process of TCH, certain inherent compounds may inhibit its diastase activity. To examine this hypothesis, the characteristic chemical constituents of TCH were further investigated.

### 3.2 Metabolomics analysis of honey

Flavonoids are the main phytochemicals present in honey, which also exhibit appreciable inhibitory activity on *α*-amylase in many studies (Li, Zhang, et al., 2023; Wang et al., 2025). To further explore the potential correlation between flavonoids in TCH and low diastase activity, this study utilized LC-MS/MS to conduct a comprehensive analysis of the composition and content of flavonoids in TCH, TCN, and 11 other honeys.

**Fig. 2:**
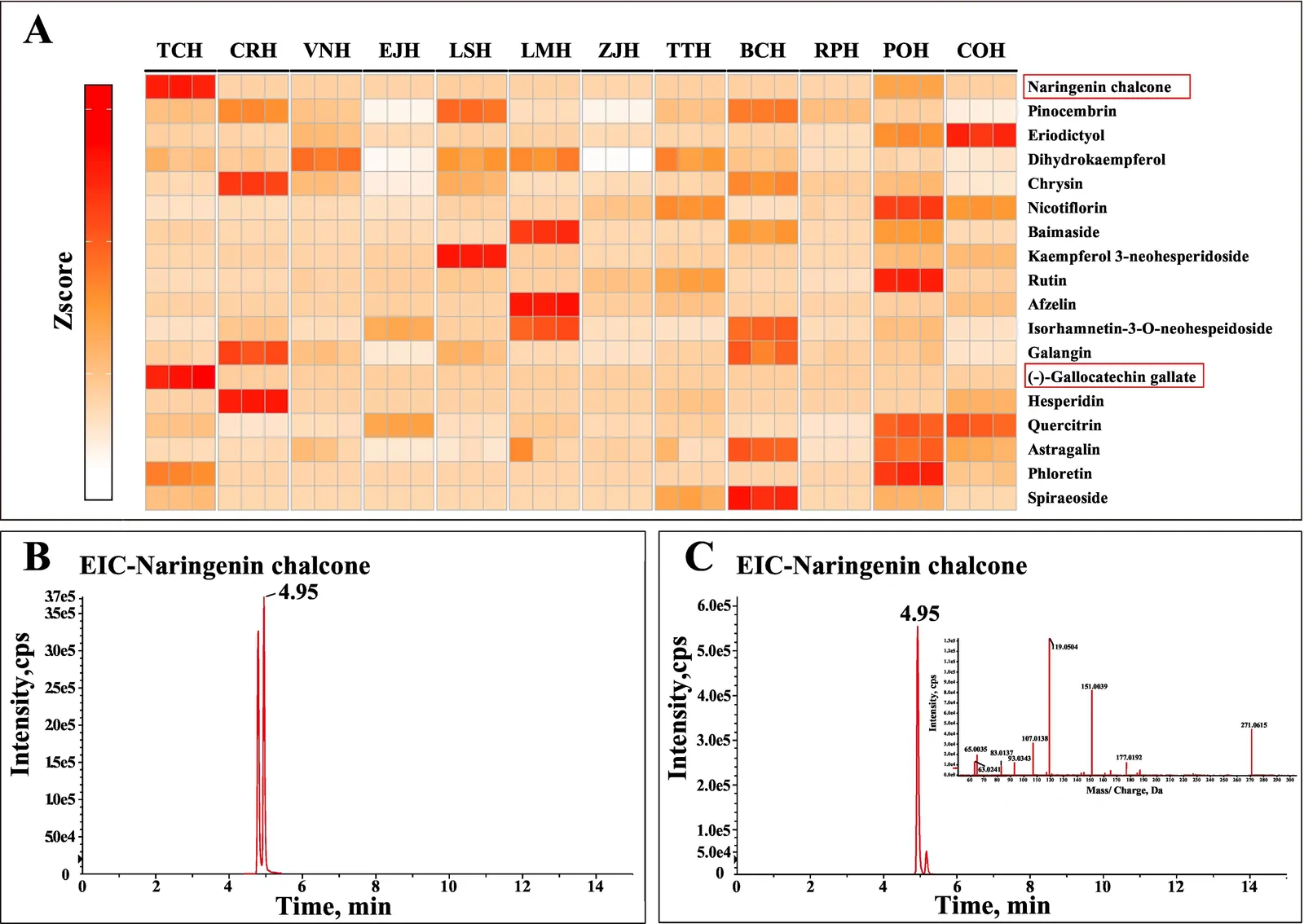
LC-MS/MS analysis of honey. (A) Heatmap analysis of the 18 types of nectar-source flavonoids in TCH and 11 other common commercial honey. (B) Extracted ion chromatogram (EIC) of naringenin chalcone (NGC) standard. **(**C) EIC and tandem mass spectrum of NGC in TCH.

All the total ion chromatograms (TIC) were shown in Fig. S2. A total of 35 types of flavonoids were identified in TCH (Table S3), while 34 were detected in TCN (Table S4). Flavonoids in honey are mainly derived from nectar plants (Li, Huang, et al., 2023), there were 18 flavonoids shared between TCH and TCN. As shown in Table 1, naringenin chalcone (NGC) was the most abundant flavonoid in TCH with content of 872.23 ± 31.18 μg/g, followed by Pinocembrin and Baimaside, with content of 454.93 ± 13.5 μg/g and 107.18 ± 4.44 μg/g respectively. Heatmap analysis was conducted to visualize the content distribution of the 18 types of nectar-source flavonoids in TCH and 11 other honeys. As shown in Fig. 2A, the NGC contents in TCH were significantly higher than those in 11 other honeys. Therefore, NGC is a characteristic high-content flavonoid that differentiated TCH from 11 other honeys. Meanwhile, (−)-gallocatechin gallate (GCG) was the unique flavonoid marker of TCH and was not detected in the other 11 honeys (Jiang et al., 2024). The chromatographic retention time and MS spectra of identified NGC in TCH were the same as NGC standard (Fig. 2B and C), which confirmed the accurate identification.

**Table 1:** 18 flavonoids in *Triadica cochinchinensis* honey derived from its nectar.

No.	Compounds	Molecular Weight	Formula	Content (ng/g honey)
1	Naringenin chalcone	272.07	C_15_H_12_O_5_	872.23 ± 31.18
2	Pinocembrin	256.07	C_15_H_12_O_4_	454.93 ± 13.50
3	Eriodictyol	288.06	C_15_H_12_O_6_	16.51 ± 3.15
4	Dihydrokaempferol	288.06	C_15_H_12_O_6_	22.86 ± 2.95
5	Chrysin	254.06	C_15_H_10_O_4_	64.06 ± 5.05
6	Nicotiflorin	594.16	C_27_H_30_O_15_	10.01 ± 1.03
7	Baimaside	626.15	C_27_H_30_O_17_	107.18 ± 4.44
8	Kaempferol 3-neohesperidoside	594.16	C_27_H_30_O_15_	63.93 ± 2.27
9	Rutin	610.15	C_27_H_30_O_16_	7.66 ± 0.54
10	Afzelin	432.11	C_21_H_20_O_10_	3.67 ± 0.30
11	Isorhamnetin-3-O-neohespeidoside	624.17	C_28_H_32_O_16_	1.94 ± 0.17
12	Galangin	270.05	C_15_H_10_O_5_	18.45 ± 2.97
13	(−)-Gallocatechin gallate	458.08	C_22_H_18_O_11_	57.58 ± 4.28
14	Hesperidin	610.19	C_28_H_34_O_15_	9.71 ± 2.12
15	Quercitrin	448.10	C_21_H_20_O_11_	40.03 ± 2.22
16	Astragalin	448.10	C_21_H_20_O_11_	4.52 ± 0.32
17	Phloretin	274.08	C_15_H_14_O5	4.41 ± 0.35
18	Spiraeoside	464.10	C_21_H_20_O_12_	4.45 ± 0.50

In previous studies, NGC exhibited α-amylase inhibitory activity by occupying enzyme active center, thereby preventing the binding of substrate (Masson & Mukhametgalieva, 2023), GCG also exerts potent inhibition on α-amylase by forming enzyme complexes that induce enzyme conformational changes (Wu et al., 2018). Therefore, NGC (high content in TCH) and GCG (unique in TCH) were identified as the key characteristic flavonoids in TCH that may contribute to the low diastase activity. Subsequent studies were focused on these two flavonoids with in vitro and in vivo experiments.

### 3.3 Inhibitory effects of GCG and NGC on α-amylase

#### 3.3.1 Inhibitory activity of GCG and NGC on α-amylase

**Fig. 3:**
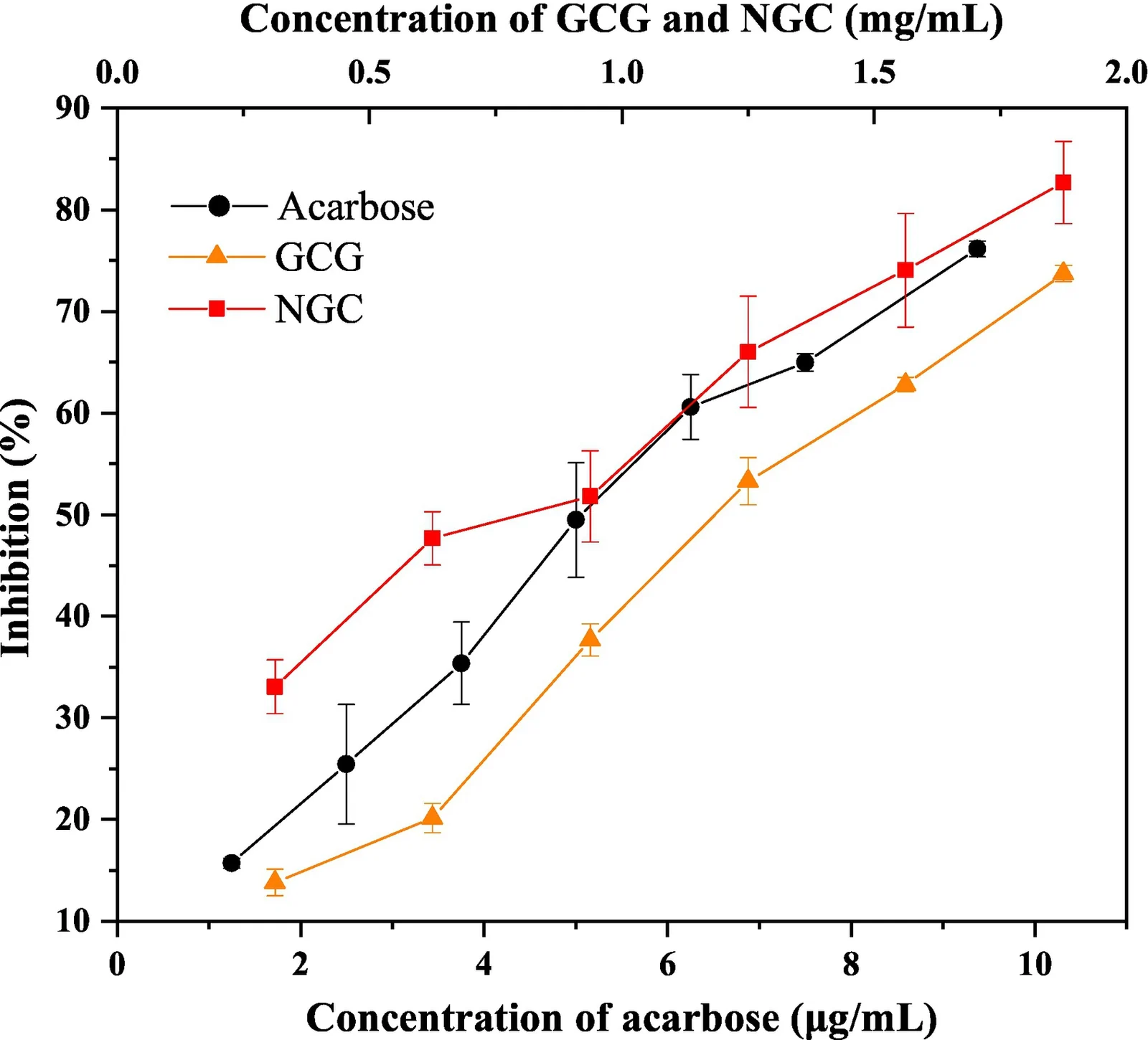
Inhibitory activity of (−)-gallocatechin gallate (GCG) and NGC against α-amylase.

The principle of α-amylase activity determination is as follows: corn starch is hydrolyzed by α-amylase to generate reducing sugars, which can react with DNS under heating conditions to form a brown-red product with the maximum absorption around 540 nm (Wei et al., 2025). Therefore, by fixing the reaction time, the absorbance increase at 540 nm of the reaction system can reflect the relative activity of α-amylase in hydrolyzing corn starch. As shown in Fig. 3, both GCG and NGC exhibited significant inhibitory effects on α-amylase hydrolysis activity, and the effects were concentration-dependent. According to the dose-inhibition activity relationship curve, the half-maximal inhibitory concentrations (IC50) of GCG and NGC on α-amylase in the reaction system were approximately 1.25 and 0.75 mg/mL, respectively. Acarbose was used as a positive control with IC50 around 5 μg/mL. GCG belongs to the flavan-3-ol category of flavonoids. Previous research has shown that flavan-3-ols have higher affinity to PPA than amylose and maltopentaose (Desseaux et al., 2018). The galloyl moieties of GCG strengthen its inhibitory effect against human salivary *α*-amylase (Zhu et al., 2020). The IC50 of GCG on human salivary *α*-amylase was 24 μg/mL, which is lower than our result (Yilmazer-Musa et al., 2012). It might be attributed to differences in enzyme sources and reaction systems. NGC, as a chalcone, also represents a novel class of α-amylase inhibitors due to the C2=C3 in C ring and polyhydroxylated structure (Ali et al., 2021; Martinez-Gonzalez et al., 2019). During the maturation process of immature TCH, its moisture content progressively decreases (Li et al., 2022). Consequently, the increase of GCG and NGC concentrations enhance the inhibitory effects on diastase activity in TCH. To characterize the inhibitory effects of GCG and NGC, their inhibition kinetics were then examined.

#### 3.3.2 Inhibition kinetics of GCG and NGC on α-amylase

**Fig. 4:**
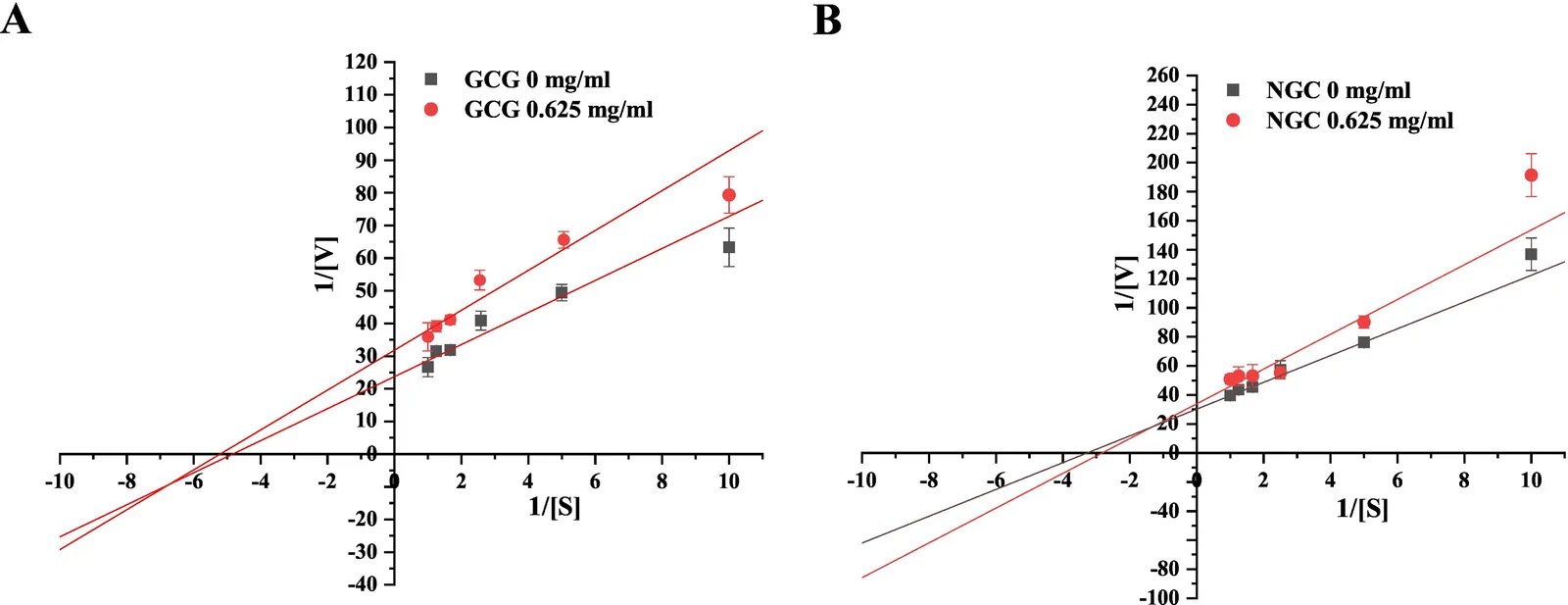
Inhibition kinetics of α-amylase by GCG (A) and NGC (B).

To explain the interaction between enzymes and inhibitors, four types of reversible inhibition are commonly recognized: competitive inhibition, non-competitive inhibition, uncompetitive inhibition, and mixed-type inhibition (Masson & Mukhametgalieva, 2023). In this study, the classical Lineweaver-Burk method was used to analyze the inhibition kinetics of GCG and NGC on α-amylase. The catalytic activity of α-amylase under a series of substrate concentrations was determined, and the Lineweaver-Burk double reciprocal curves were plotted. As shown in Fig. 4, with inhibitor concentrations of 0 and 0.625 mg/mL, the two double reciprocal curves of GCG intersected in the third quadrant, while the two curves of NGC intersected in the second quadrant. The results indicated that GCG and NGC act as reversible inhibitors of α-amylase through a mixed-type inhibition, which is consistent with the findings of Proença et al. (2021) that GCG and chalcones frequently exhibit mixed-type inhibition against α-amylase. This suggested that GCG and NGC can bind to both free enzyme and substrate-enzyme complexes, thereby decreasing the reaction rate (Picot et al., 2014; Saboury, 2009). Their binding interactions with α-amylase were subsequently performed by fluorescence quenching and molecular docking analyses.

### 3.4 Binding of GCG and NGC to α-amylase

#### 3.4.1 Fluorescence quenching of α-amylase by GCG and NGC

**Fig. 5:**
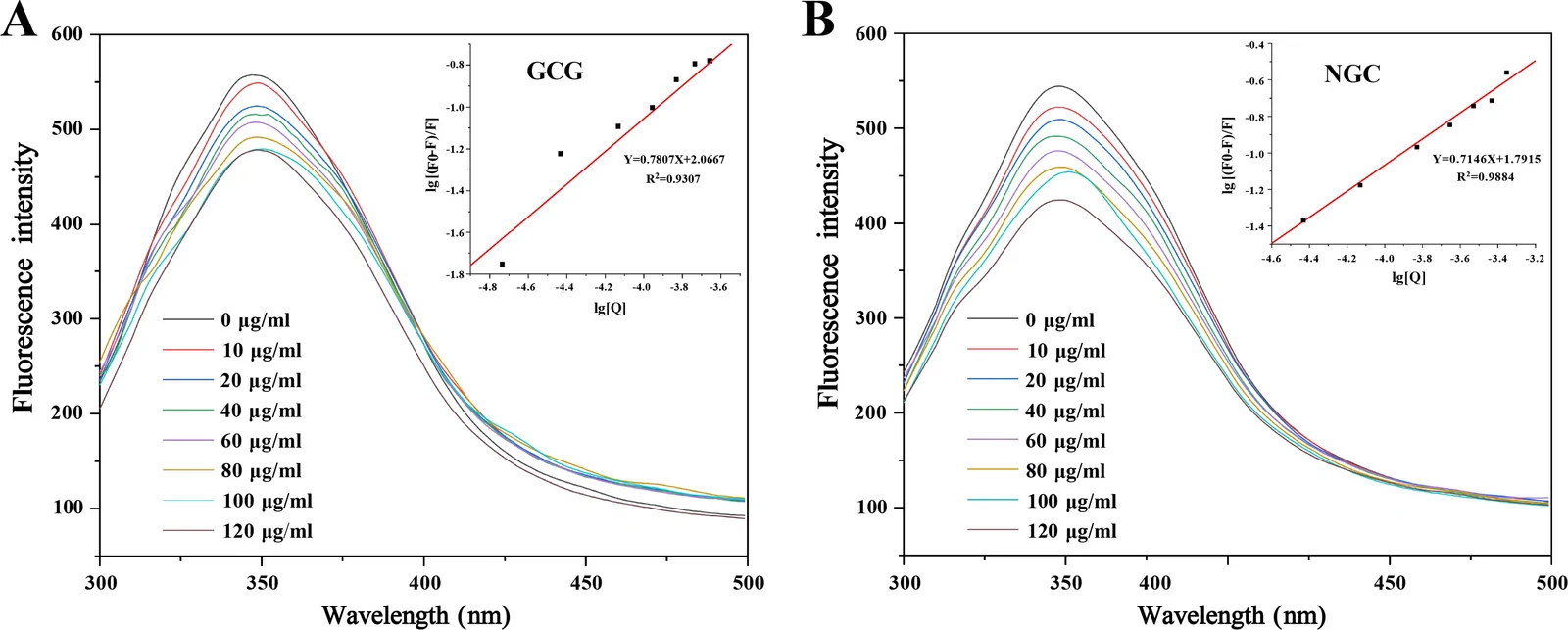
Fluorescence spectra of GCG (A) and NGC (B) binding to α-amylase.

Proteins contain aromatic amino acids such as tryptophan and tyrosine, which can emit fluorescence (Guo et al., 2011). However, when small molecules bind to the enzyme protein, they may change the intramolecular microenvironment of protein, thereby causing the quenching of its intrinsic fluorescence. Therefore, the interaction between GCG and NGC and α-amylase was studied using fluorescence titration. As shown in Fig. 5, at an excitation wavelength of 280 nm, the maximum emission wavelength of α-amylase is around 350 nm. Adding increasing concentrations of GCG (Fig. 5A) and NGC (Fig. 5B) to the enzyme solution, a gradually quenching of the intrinsic fluorescence of α-amylase was observed, indicating the binding of inhibitor to the enzyme protein. Moreover, the wavelength of maximum fluorescent intensity exhibited a slight red shift, indicating that the binding of the GCG and NGC can change the hydrophobic microenvironment of amino acid in α-amylase, thereby making the spatial structure of the enzymes more flexible (Fei et al., 2014).

The fluorescence quenching process can be described by the Stern-Volmer equation (Zhou et al., 2021):

(3) $$\frac{F_{0}}{F}=1+K_{\mathrm{SV}}\left[Q\right]=1+\mathrm{Kq\tau}₀\left[Q\right]$$

(4) $$\mathrm{Kq}=\frac{K_{\mathrm{SV}}}{\tau ₀}$$

In the formula, F_0_ represents the intrinsic fluorescence intensity of α-amylase, F is the fluorescence intensity after inhibitor adding, Kq is the rate constant of the quenching process, τ_0_ is the lifetime of the fluorophore (usually 1 × 10^−8^ s), [Q] is the concentration of the inhibitor, and K_SV_ is the Stern-Volmer quenching constant. Generally, the Kq value of static fluorescence quenching process is greater than 2.0 × 10^10^ L/(mol·s) (Shi et al., 2010). In this study, the Kq values of GCG and NGC were calculated as 7.65 × 10^10^ L/(mol·s) and 6.40 × 10^10^ L/(mol·s), respectively. Therefore, the interaction between GCG or NGC and α-amylase is a static quenching process. Static quenching is characterized by the formation of a ground-state complex between quencher and fluorophore. This mechanism has been observed between PPA and flavonoids (Martinez-Gonzalez et al., 2019).

For static quenching, the binding of the ligand to the macromolecule can be described by eq. (5) (Zhou et al., 2021), where Ka is the binding constant, and n is the number of binding sites.

(5) $$\mathrm{lg}\frac{F_{0}-F}{F}=n\mathrm{lg}\left[Q\right]+\mathrm{lg}\mathrm{Ka}$$

Based on the results of linear regression, the values of lgKa and n for GCG were calculated as 2.07 and 0.78, respectively, while the values for NGC were 1.79 and 0.71 respectively. The n value close to 1 indicates that the combinations of GCG and NGC with PPA showed a theoretical one binding site, implying that the binding interactions occur mainly at the enzyme catalytic active site (Lim et al., 2022). The n values for the binding of flavonoids from dandelion to α-amylase were also reported to be close to 1 (Huang et al., 2021).

#### 3.4.2 Molecular docking simulation of GCG and NGC binding to α-amylase

**Fig. 6:**
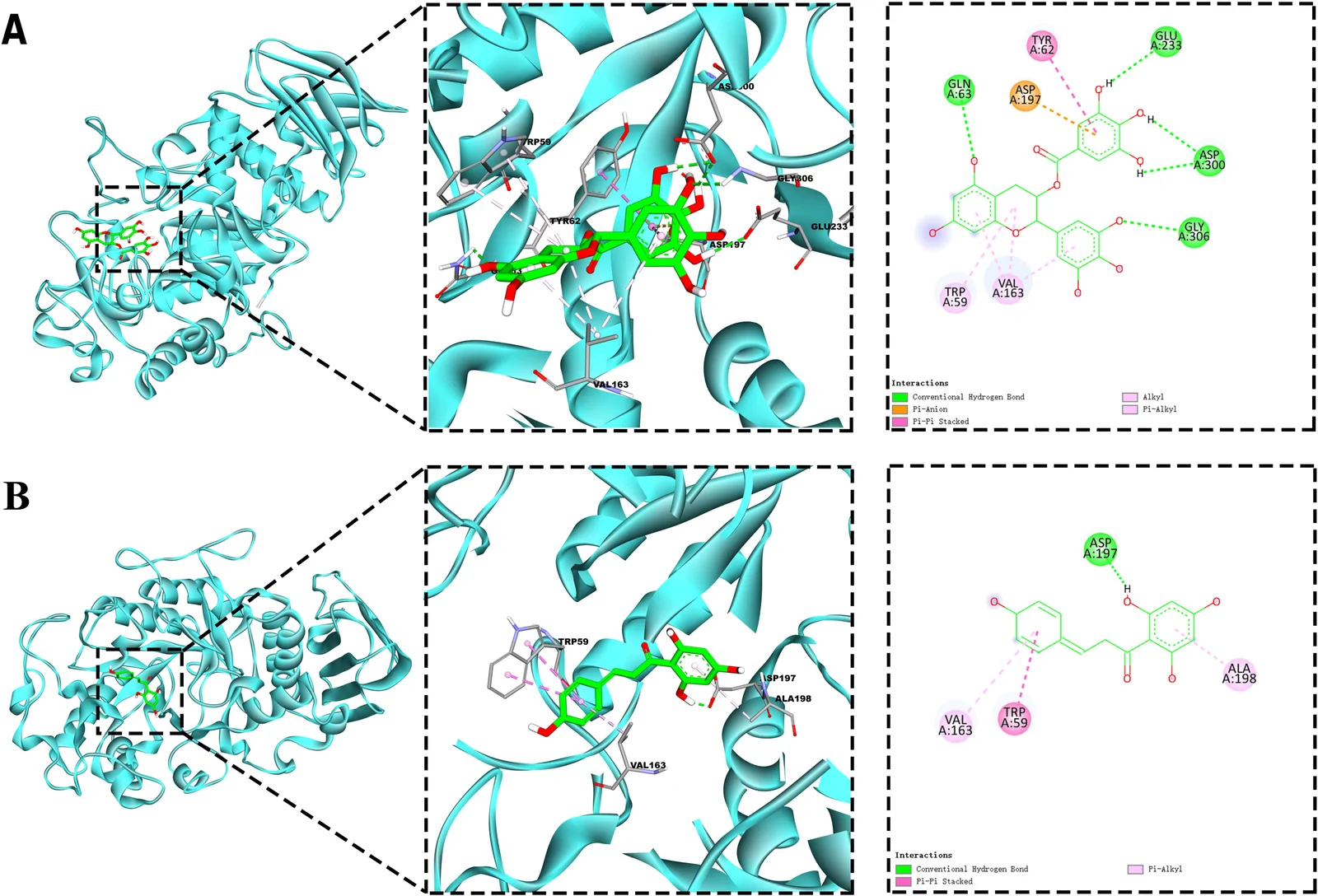
Molecular docking simulations of GCG (A) and NGC (B) with α-amylase.

To better understand the binding between GCG and NGC and α-amylase, molecular docking simulations were conducted. α-Amylase has three regions termed A, B, and C and the active site is in the largest region A (Rasouli et al., 2017). As shown in Fig. 6A, GCG can bind to α-amylase near the active center, forming hydrogen bonds with Gln63, Glu233, Asp300, and Gly306 in the A region, and forming π-π stacking and other hydrophobic interactions with Trp59, Val163, Asp197, and Tyr62. The smallest binding free energy was −8.546 kcal/mol. Asp197, Glu233, and Asp300 are the three key amino acid residues of α-amylase (Rasouli et al., 2017). The formation of hydrogen bonds between GCG and these key amino acid residues will weaken the affinity between the enzyme and the substrate, thereby reducing the catalytic activity. In addition, the hydrophobic interactions of GCG with the enzyme may also cause slight changes in the enzyme's conformation, thereby affecting its catalytic activity. GCG was previously reported to interact with key amino acid residues in the active-site pocket of α-amylase and inhibit the enzyme through the mixed type (Wu et al., 2018).

In contrast, NGC formed fewer secondary bond interactions with α-amylase (Fig. 6B), including one hydrogen bond with Asp197, and hydrophobic interactions with Trp59, Ala198, and Val163. Correspondingly, the binding free energy of NGC with α-amylase was −8.222 kcal/mol, indicating a slightly lower binding affinity than that of GCG. This difference may be attributed to the absence of a galloyl group and fewer hydroxyl groups in NGC relative to GCG, which is consistent with previous findings that specific gallate esters and phenolic hydroxyl groups enhance the α-amylase inhibitory activity and structural disruption (Wen et al., 2025). Overall, the molecular modeling results showed that the GCG and NGC specifically bind to important positions with largest region A of α-amylase, which is in-line with their mixed-type inhibition kinetics and static quenching process behavior.

### 3.5 Effects of TCH, GCG, and NGC on gene relative expression of α-amylase in workers' hypopharyngeal glands

**Fig. 7:**
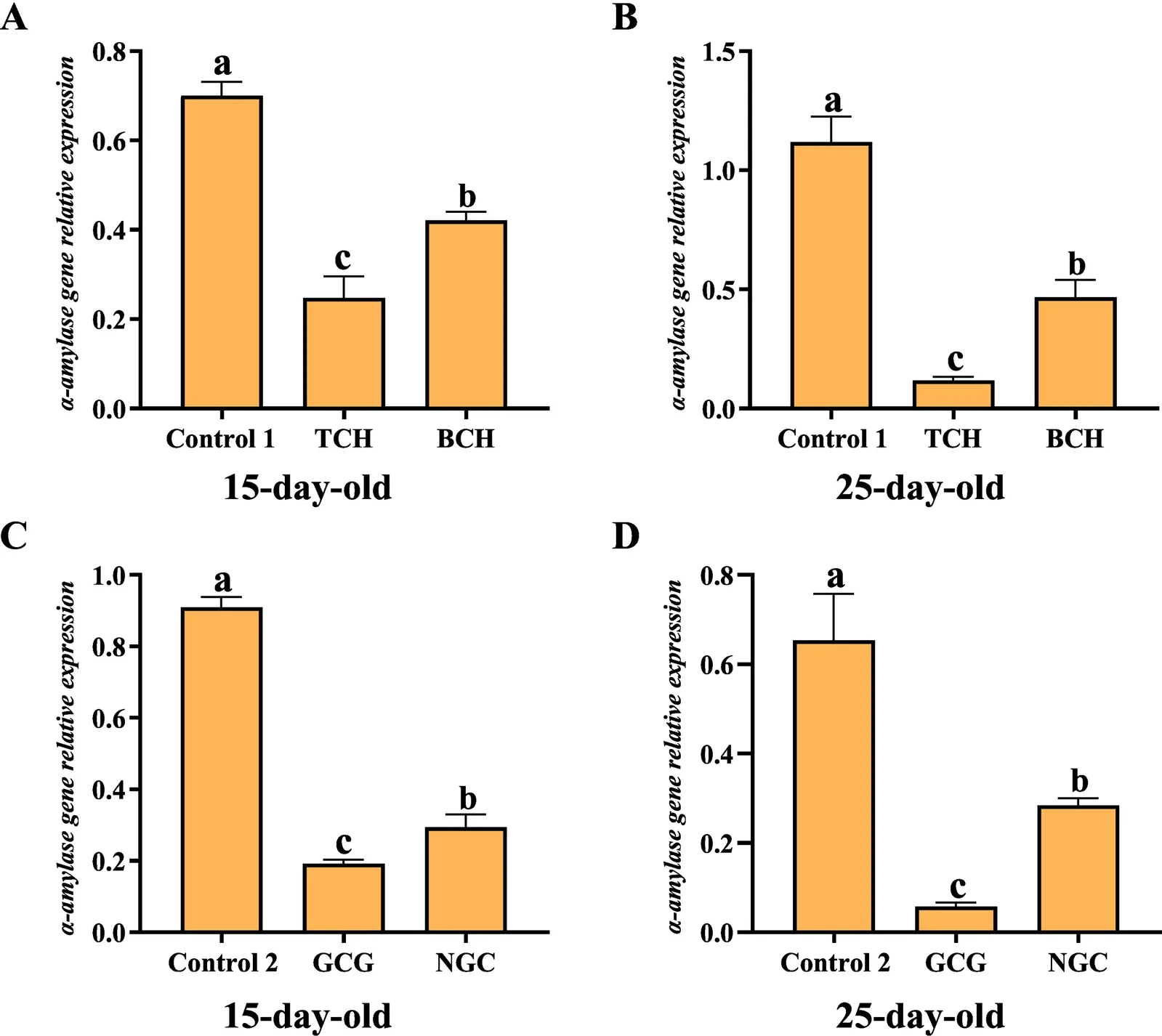
Effects of feeding honey and flavonoids on *α-amylase* gene relative expression in hypopharyngeal glands of worker bees. **(**A) 15-day-old worker bees fed 50% sucrose solution (Control 1), TCH and BCH. **(**B) 25-day-old worker bees fed 50% sucrose solution, TCH and BCH. **(**C) 15-day-old worker bees fed 50% sucrose solution with 0.3% ethanol (Control 2), (−)-gallocatechin gallate (GCG) and naringenin chalcone (NGC). (D) 25-day-old worker bees fed 50% sucrose solution with 0.3% ethanol, GCG and NGC. Data were analyzed using one-way ANOVA, followed by Tukey's HSD post hoc test. Different lowercase letters indicated significant differences (*P* < 0.05).

In vitro enzymatic results showed that GCG and NGC inhibited *α*-amylase activity. We next examined their effects on *α-amylase* gene expression in workers' hypopharyngeal glands by in vivo feeding experiments. As shown in Fig. 7A and B, a consistent pattern was observed in both 15-day-old honey-ripening workers and 25-day-old foragers. Compared with the worker bees fed with BCH, the relative expression of *α-amylase* gene in the hypopharyngeal glands was significantly lower in worker bees fed with TCH by 41.10% and 74.79% in15- and 25-day-old workers, respectively. Compared to TCH, BCH only contained trace amounts of NGC (22.08 ng/g), but not GCG. The presence of GCG and NGC in TCH likely resulted in lower *α-amylase* gene relative expression in worker bees fed with TCH. To further verify this speculation, worker bees were fed with sucrose solutions containing GCG or NGC. Compared with the sucrose-fed control group, *α-amylase* gene relative expression was significantly reduced by 78.92% and 67.71% in 15-day-old workers supplemented with GCG and NGC, respectively (Fig. 7C). Similarly, 91.14% and 56.48% suppressions were observed in 25-day-old workers from the two groups, respectively (Fig. 7D). It was thus evident that the presence of GCG and NGC in TCH leads to reducing *α-amylase* gene relative expression in worker bees consuming this honey type. Similar findings have been reported in *Drosophila* model. Dietary supplementation with bayberry leaf proanthocyanidins (flavonoids) reduced *α-amylase* gene expression in *Drosophila melanogaster* (Wang et al., 2022)*.*

In a typical honey bee colony, 15-day-old workers are primarily engaged in honey ripening and wax secretion, whereas 25-day-old workers mainly undertake nectar foraging tasks (Ament et al., 2010; Johnson, 2003). Both age groups are critically involved in nectar collection or processing. During foraging, foragers add hypopharyngeal-gland-derived enzymes including diastase and invertase, to the nectar, while honey-ripening bees contribute to their secreted enzymes during the in-hive honey ripening (Ahmad et al., 2021; Qi et al., 2015). Therefore, the reduced *α-amylase* expression in both age workers may decrease the contribution of bee-derived diastase during nectar collection and ripening, which partly results in the low diastase activity in TCH.

In addition, worker bees in the sucrose control group exhibited significantly higher *α-amylase* gene relative expression than those fed either TCH or BCH. It should also be noted that the control diet contained 50% sucrose, whereas the sucrose content in both TCH and BCH diets was less than 5%. The high sucrose level in the control diet may have stimulated *α-amylase* gene relative expression in the hypopharyngeal glands, possibly due to the greater need for enzymatic conversion of sucrose into glucose and fructose (Ueno et al., 2009; Ueno et al., 2015).

### 3.6 Network pharmacological analysis

**Fig. 8:**
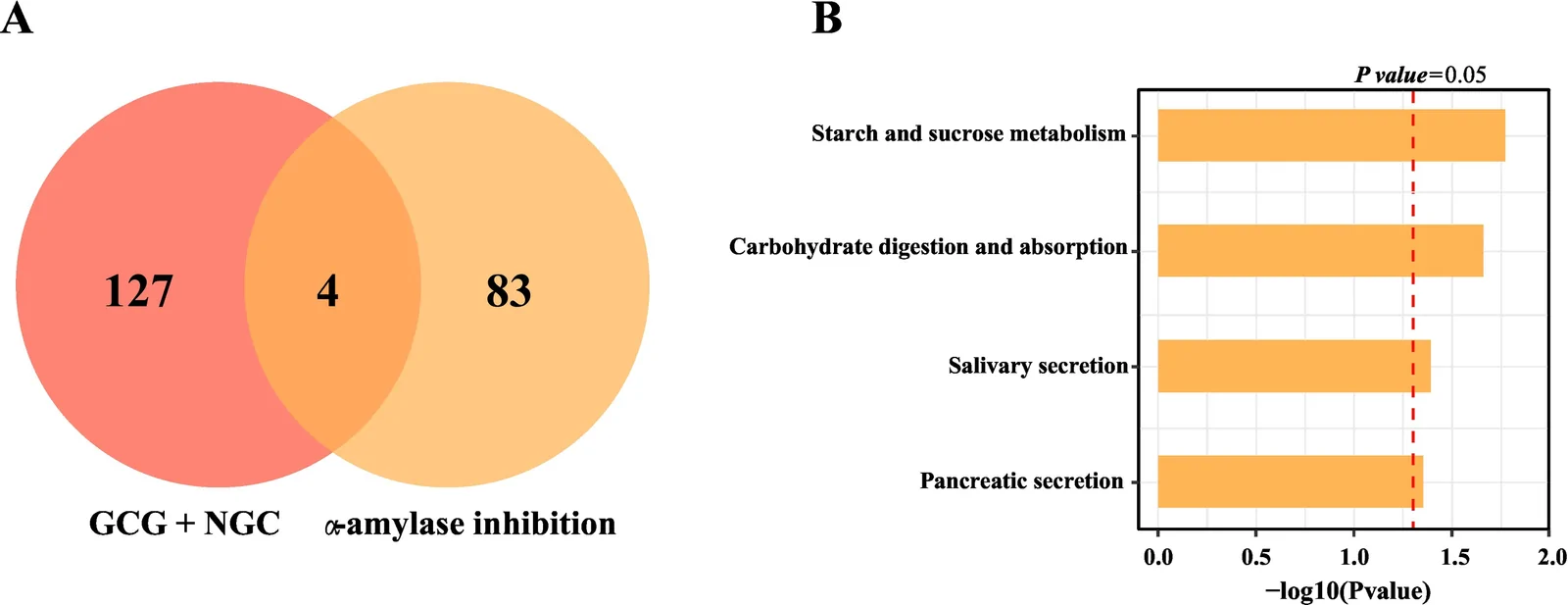
Network pharmacological analysis of GCG and NGC in α-amylase inhibition. **(**A) Venn diagram of shared targets between GCG and NGC and α-amylase inhibition. (B) KEGG enrichment analysis.

To investigate the potential mechanism by which NGC and GCG inhibited *α-amylase* gene expression in worker bees, network pharmacology was utilized to predict their potential targets of NGC and GCG, and a total of 131 targets were identified. Meanwhile, 87 α-amylase inhibition- related genes were obtained (Fig. 8A). Venn analysis revealed four core potential targets at the intersection of NGC and GCG and α-amylase inhibition- related genes: *AMY1A、VEGFA、BCL2* and *PTPN1*. *AMY1A* is a member of the *α-amylase* gene cluster in human genome and has been positively correlated with α-amylase expression in human saliva (Fernández & Wiley, 2017). *AMY1A* and the *α-amylase* gene in honey bees are highly conserved and homologous genes.

Further KEGG enrichment analysis was performed on these four targets. Four significantly enriched pathways that related to α-amylase were shown in Fig. 8B. α-amylase is a key digestive enzyme and the most abundant protein in human saliva and pancreas (Boehlke et al., 2015; Yin et al., 2023). Salivary secretion pathway is associated with the synthesis and secretion of α-amylase, which is modulated by crosstalk between fluid and protein secretory pathways via cAMP/protein kinase A (Song et al., 2024). The pancreatic secretion pathway can also regulate pancreatic acinar cells to synthesize and secrete α-amylase (Guo et al., 2020). α-amylases hydrolyze the linear-1,4 glycosidic bonds in the starch (amylose and amylopectin) to produce linear glucose oligomers (Farazi et al., 2024). Carbohydrate digestion and absorption and starch and sucrose metabolism are the core metabolic pathways for the hydrolytic function of α-amylase (Park, 2008). The results suggested that NGC and GCG may reduce the *α-amylase* gene expression in worker bees by targeting the homologous genes *AMY1A*, *VEGFA*, *BCL2* and *PTPN1* in worker bees, thereby interfering with the four α-amylase-related pathways.

## 4 Conclusions

This study revealed the underlying mechanisms of the naturally low diastase activity in TCH, which may be associated with its flavonoid constituents. LC-MS/MS analysis identified that GCG (unique) and NGC (highest content) were the two characteristic flavonoids in TCH. These flavonoids exerted a dual inhibition on diastase activity in TCH. First, GCG and NGC exhibited direct inhibitory activity on α-amylase activity via mixed-type inhibition. Second, GCG and NGC supplementation downregulated the expression of *α-amylase* gene in the hypopharyngeal glands of worker bees, which might be associated with four α-amylase-related pathways. These findings offer scientific insights into the naturally low diastase activity in TCH and contribute to the establishment of TCH-specific quality evaluation standards.

## CRediT authorship contribution statement

**Huizhi Jiang:** Writing – review & editing, Writing – original draft, Formal analysis, Data curation, Conceptualization. **Zhen Li:** Writing – original draft, Methodology, Conceptualization. **Tianyu Xu:** Writing – original draft, Formal analysis, Data curation. **Min Xu:** Validation, Data curation. **Weixuan Chen:** Visualization, Validation. **Shengyang He:** Software, Investigation. **Xiaobo Wu:** Software, Resources. **Weiyu Yan:** Visualization, Investigation. **Qingfeng Zhang:** Writing – review & editing, Visualization, Methodology. **Zhijiang Zeng:** Writing – review & editing, Writing – original draft, Supervision, Resources, Project administration, Funding acquisition.

## Declaration of competing interest

The authors declare that they have no known competing financial interests or personal relationships that could have appeared to influence the work reported in this paper.

## Data Availability

Data will be made available on request.
