# Supplementary material for: Flavonoid constituents contribute to the low diastase activity of Triadica cochinchinensis honey through in vitro enzyme inhibition and in vivo transcriptional regulation
Source: Food Chem X. 2026 Sep 12;39:104452. doi: 10.1016/j.fochx.2026.104452 (PMC13595074; doi:10.1016/j.fochx.2026.104452)
Supplement: Supplementary file 1 [file mmc1.pdf]

Flavonoid constituents contribute to the low diastase activity of *Triadica cochinchinensis* honey through *in vitro* enzyme inhibition and *in vivo* transcriptional regulation

Huizhi Jiang, Zhen Li, Tianyu Xu, Min Xu, Weixuan Chen, Shengyang He, Xiaobo Wu, Weiyu Yan, Qingfeng Zhang *, Zhijiang Zeng *

Table S1 Native pollen rate of *Triadica cochinchinensis* honey and 10 types of Chinese common commercial monofloral honey.

Honey Variety	Native pollen rate (%)	Honey Variety	Native pollen rate (%)
Triadica cochinchinensis honey (TCH)	82.08 ± 2.55	*Brassica campestris* honey (BCH)	84.58 ± 3.63
Vitex negund honey (VNH)	75.48 ± 4.30	*Robinia pseudoacacia* honey (RPH)	74.67 ± 4.07
Ziziphus jujuba honey (ZJH)	79.73 ± 5.17	*Tilia tuan* honey (TTH)	82.72 ± 4.86
Eriobotrya japonica honey (EJH)	83.64 ±4.13	*Litchi chinensis* Sonn honey (LSH)	86.30 ± 1.48
Citrus reticulata honey (CRH)	75.34 ± 2.46	*Lycium chinense* Miller honey (LMH)	78.98 ± 4.01
Camellia oleifera honey (COH)	86.96 ± 4.33		

Table S2 Primers are used in quantitative PCR and their sequences.

Target gene	Forward primer（5'-3'）	Reverse primer (5'-3')
α-Amylase	GATGTTCCGGATTTGTCGCG	CACGTCGCAATATTGGCCAG
gapdh	GCTGGTTTCATCGATGGTTT	ACGATTTCGACCACCGTAAC

Table S3 35 flavonoids identified in *Triadica cochinchinensis* Honey (TCH).

No	Compounds	Class	Molecular Weight	Formula
1	Neohesperidin	Flavanones	610.19	$C_{28}H_{34}O_{15}$
2	Oroxin A	Flavones	432.11	$C_{21}H_{20}O_{10}$
3	Dihydromyricetin	Flavanonols	320.05	$C_{15}H_{12}O_{8}$
4	Nicotiflorin	Flavones	594.16	$C_{27}H_{30}O_{15}$
5	Kaempferol 3-neohesperidoside	Flavonols	594.16	$C_{27}H_{30}O_{15}$
6	Isorhamnetin-3-O-neohespeidoside	Flavonols	624.17	$C_{28}H_{32}O_{16}$
7	Taxifolin	Flavanonols	304.06	$C_{15}H_{12}O_{7}$
8	Rutin	Flavonols	610.15	$C_{27}H_{30}O_{16}$
9	Chrysin	Flavones	254.06	$C_{15}H_{10}O_{4}$
10	Mangiferin	Xanthones	422.08	$C_{19}H_{18}O_{11}$
11	Galangin	Flavones	270.05	$C_{15}H_{10}O_{5}$
12	Genistin	Isoflavanones	432.11	$C_{21}H_{20}O_{10}$
13	(-)-Gallocatechin gallate	Flavanols	458.08	$C_{22}H_{18}O_{11}$
14	(-)-Epigallocatechin	Flavanols	306.07	$C_{15}H_{14}O_{7}$
15	Hesperidin	Flavanones	610.19	$C_{28}H_{34}O_{15}$
16	Pinocembrin	Flavanones	256.07	$C_{15}H_{12}O_{4}$
17	Quercitrin	Flavonols	448.10	$C_{21}H_{20}O_{11}$
18	Astragalin	Flavonols	448.10	$C_{21}H_{20}O_{11}$
19	Eriodictyol	Flavanones	288.06	$C_{15}H_{12}O_{6}$
20	Alpinetin	Flavanones	270.09	$C_{16}H_{14}O_{4}$
21	Baimaside	Flavonols	626.15	$C_{27}H_{30}O_{17}$
22	Dihydrokaempferol	Flavanonols	288.06	$C_{15}H_{12}O_{6}$
23	Isosakuranin	Flavanones	448.14	$C_{22}H_{24}O_{10}$
24	(-)-Catechin gallate	Flavanols	442.09	$C_{22}H_{18}O_{10}$
25	Sinensetin	Flavones	372.12	$C_{20}H_{20}O_{7}$
26	Vitexin	Flavone glycosides	432.11	$C_{21}H_{20}O_{10}$
27	Isosakuranetin	Flavanones	286.08	$C_{16}H_{14}O_{5}$
28	5,7,3',4'-Tetramethoxyflavone	Flavones	342.11	$C_{19}H_{18}O_{6}$
29	Afzelin	Flavonols	432.11	$C_{21}H_{20}O_{10}$
30	Naringenin-7-glucoside	Flavanones	434.12	$C_{21}H_{22}O_{10}$
31	Phloretin	Chalcones	274.08	$C_{15}H_{14}O_{5}$
32	Myricitrin	Flavonols	464.10	$C_{21}H_{20}O_{12}$
33	Spiraeoside	Flavonols	464.10	$C_{21}H_{20}O_{12}$
34	Naringenin chalcone	Chalcones	272.07	$C_{15}H_{12}O_{5}$
35	Sieboldin	Chalcones	452.13	$C_{21}H_{24}O_{11}$

Table S4 34 flavonoids identified in *T. cochinchinensis* nectar (TCN).

No	Compounds	Class	Molecular Weight	Formula
1	Phloretin	Chalcones	274.08	$C_{15}H_{14}O_5$
2	Naringenin chalcone	Chalcones	272.07	$C_{15}H_{12}O_5$
3	Phlorizin	Chalcones	436.14	$C_{21}H_{24}O_{10}$
4	(-)-Epicatechin	Flavanols	290.08	$C_{15}H_{14}O_6$
5	Afzelechin	Flavanols	274.08	$C_{15}H_{14}O_5$
6	(-)-Gallocatechin gallate	Flavanols	458.08	$C_{22}H_{18}O_{11}$
7	(-)-Catechin	Flavanols	290.08	$C_{15}H_{14}O_6$
8	Hesperidin	Flavanones	610.19	$C_{28}H_{34}O_{15}$
9	Pinocembrin	Flavanones	256.07	$C_{15}H_{12}O_4$
10	Eriodictyol	Flavanones	288.06	$C_{15}H_{12}O_6$
11	Dihydrokaempferol	Flavanonols	288.06	$C_{15}H_{12}O_6$
12	Luteolin	Flavones	286.05	$C_{15}H_{10}O_6$
13	Galangin	Flavones	270.05	$C_{15}H_{10}O_5$
14	Chrysin	Flavones	254.06	$C_{15}H_{10}O_4$
15	Tricin	Flavones	330.07	$C_{17}H_{14}O_7$
16	Apigenin	Flavones	270.05	$C_{15}H_{10}O_5$
17	Nicotiflorin	Flavones	594.16	$C_{27}H_{30}O_{15}$
18	Scutellarin	Flavones	462.08	$C_{21}H_{18}O_{12}$
19	Kaempferol 3-neohesperidoside	Flavonols	594.16	$C_{27}H_{30}O_{15}$
20	Baimaside	Flavonols	626.15	$C_{27}H_{30}O_{17}$
21	Quercitrin	Flavonols	448.10	$C_{21}H_{20}O_{11}$
22	Rutin	Flavonols	610.15	$C_{27}H_{30}O_{16}$
23	Quercetin	Flavonols	302.04	$C_{15}H_{10}O_7$
24	Afzelin	Flavonols	432.11	$C_{21}H_{20}O_{10}$
25	Hyperoside	Flavonols	464.10	$C_{21}H_{20}O_{12}$
26	Astragalin	Flavonols	448.10	$C_{21}H_{20}O_{11}$
27	Quercetin 3-O-(6"-galloyl)-β-D-galactopyranoside	Flavonols	616.11	$C_{28}H_{24}O_{16}$
28	Tiliroside	Flavonols	594.14	$C_{30}H_{26}O_{13}$
29	2"-O-Galloylhyperin	Flavonols	616.11	$C_{28}H_{24}O_{16}$
30	Isorhamnetin-3-O-neohespeidoside	Flavonols	624.17	$C_{28}H_{32}O_{16}$
31	Avicularin	Flavonols	434.08	$C_{20}H_{18}O_{11}$
32	Spiraeoside	Flavonols	464.10	$C_{21}H_{20}O_{12}$
33	Kaempferol	Flavonols	286.05	$C_{15}H_{10}O_6$
34	Robinin	Flavonols	182.06	$C_9H_{10}O_4$

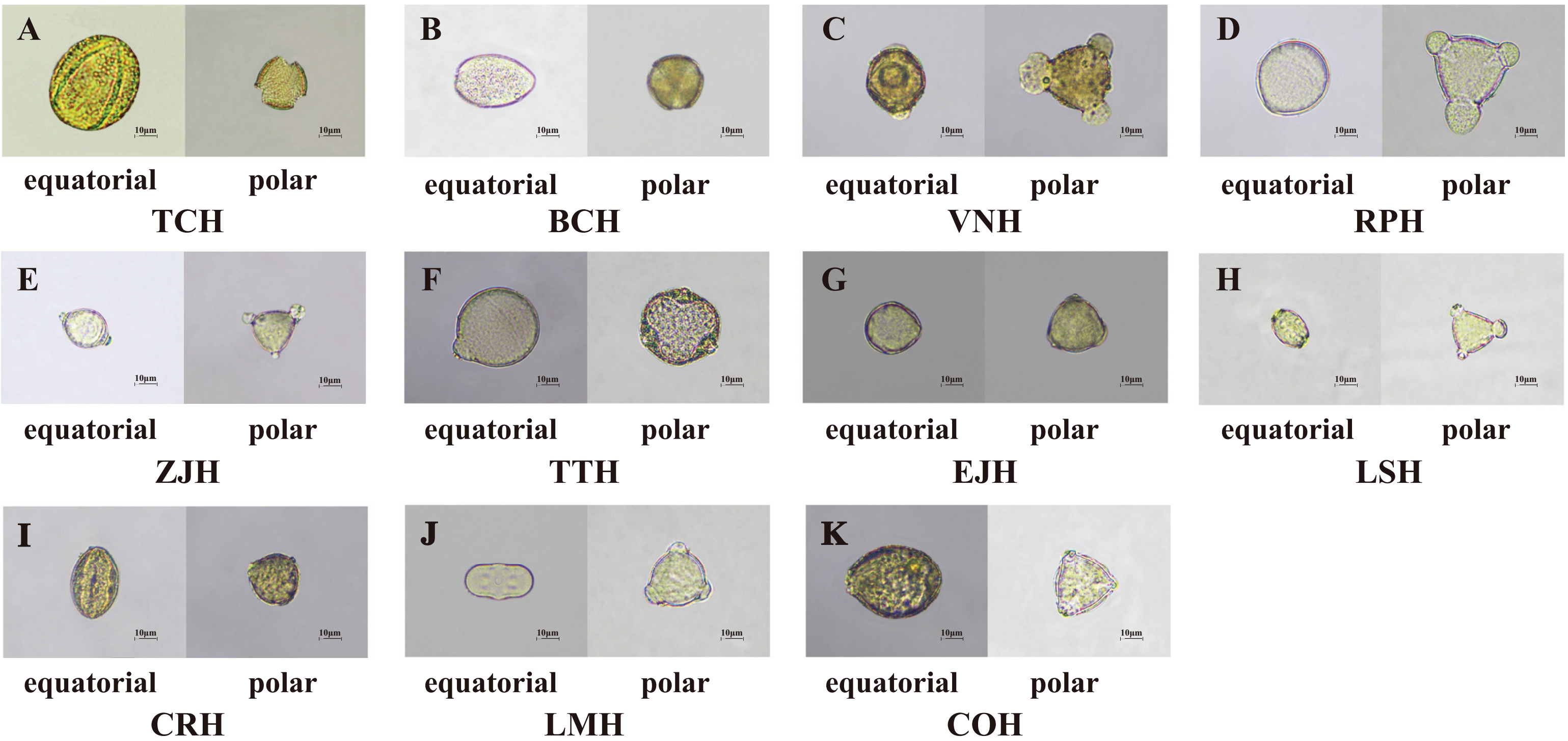

Figure S1. The pollen morphology of *Triadica cochinchinensis* honey (TCH) and other 10 common types of Chinese commercial monofloral honey.(**A**) TCH; (**B**) *Brassica campestris* honey (BCH) ; (**C**) *Vitex negundo* L. *var*. honey (VNH); (**D**) *Robinia pseudoacacia* honey (RPH); (**E**) *Ziziphus jujuba* honey (ZJH); (**F**) *Tilia tuan* honey (TTH); (**G**)*Eriobotrya japonica* honey (EJH); (**H**) *Litchi chinensis* Sonn honey (LSH); (**I**) *Citrus reticulata* honey (CRH); (**J**) *Lycium chinense* Miller honey (LMH); (**L**) *Camellia oleifera* honey(COH).

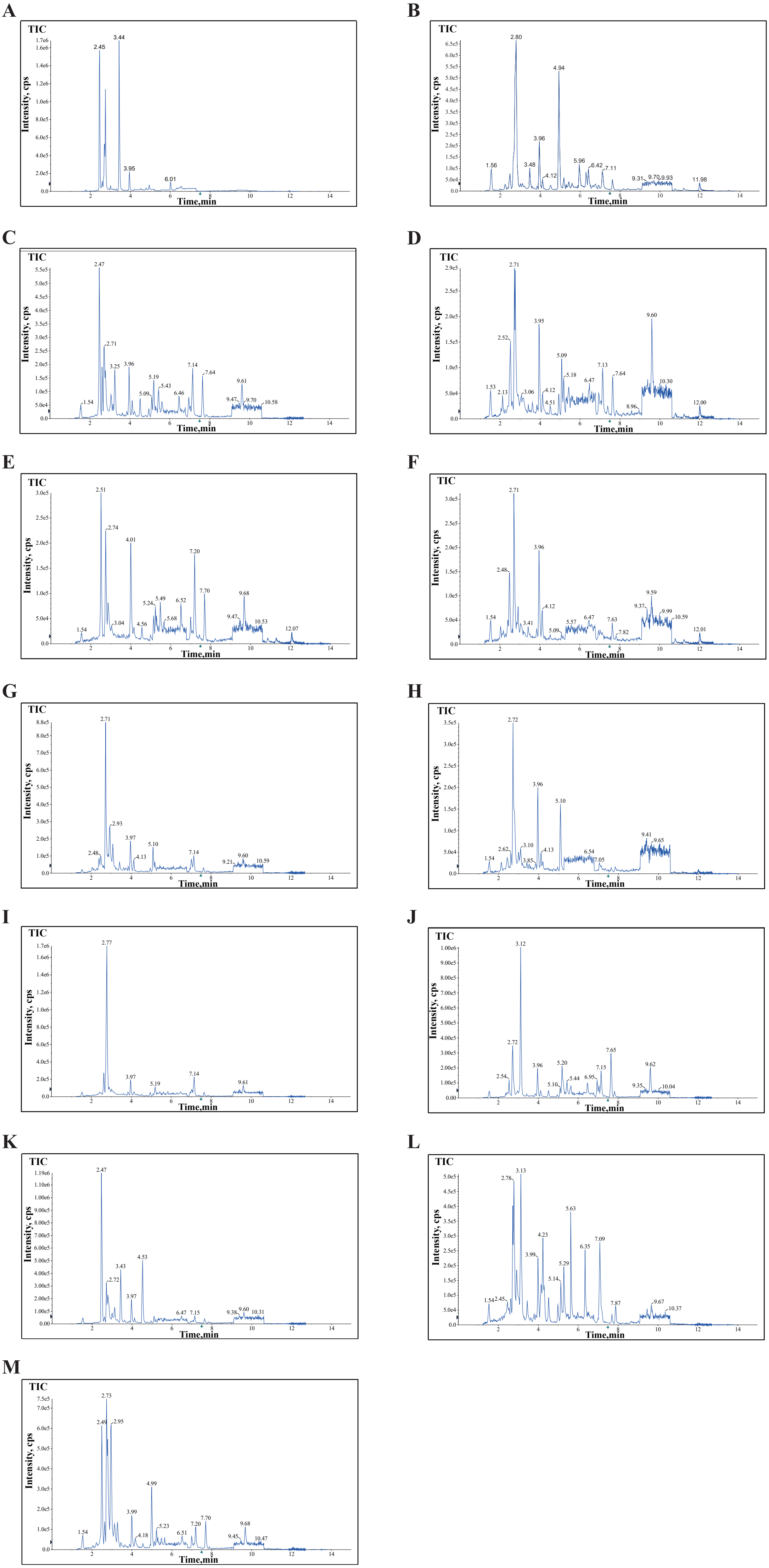
A
TIC
Intensity, cps
2.45
3.44
3.95
6.01
Time,min
B
TIC
Intensity, cps
2.80
4.94
3.96
1.56
3.48
4.12
5.96
6.42
7.11
9.31
9.70
9.93
11.98
Time,min
C
TIC
Intensity, cps
2.47
2.71
3.25
3.96
5.19
5.09
5.43
6.46
7.14
7.64
9.61
9.47
9.70
10.58
1.54
Time,min
D
TIC
Intensity, cps
2.71
2.52
3.95
9.60
5.09
5.18
7.13
7.64
6.47
10.30
1.53
2.13
3.06
4.12
4.51
8.96
12.00
Time,min
E
TIC
Intensity, cps
2.51
2.74
4.01
7.20
7.70
9.68
5.24
5.49
6.52
5.68
3.04
4.56
1.54
9.47
10.53
12.07
Time,min
F
TIC
Intensity, cps
2.71
3.96
2.48
9.59
9.37
9.99
10.59
4.12
1.54
3.41
5.09
5.57
6.47
7.63
7.82
12.01
Time,min
G
TIC
Intensity, cps
2.71
2.93
3.97
5.10
2.48
4.13
7.14
9.21
9.60
10.59
Time,min
H
TIC
Intensity, cps
2.72
3.96
5.10
9.41
9.65
2.62
3.10
4.13
3.85
1.54
6.54
7.05
Time,min
I
TIC
Intensity, cps
2.77
3.97
5.19
7.14
9.61
Time,min
J
TIC
Intensity, cps
3.12
2.72
2.54
3.96
5.20
5.10
5.44
6.95
7.15
7.65
9.62
9.35
10.04
Time,min
K
TIC
Intensity, cps
2.47
3.43
4.53
2.72
3.97
6.47
7.15
9.38
9.60
10.31
Time,min
L
TIC
Intensity, cps
3.13
2.78
5.63
4.23
3.99
7.09
6.35
5.29
5.14
1.54
2.45
7.87
9.67
10.37
Time,min
M
TIC
Intensity, cps
2.73
2.49
2.95
4.99
3.99
4.18
5.23
6.51
7.20
7.70
9.68
9.45
10.47
1.54
Time,min

Figure S2. The total ion chromatograms (TIC) of TCH, *T. cochinchinensis* nectar (TCN) and 11 other honeys. (**A**) TCN; (**B**) TCH; (**C**) BCH; (**D**) VNH; (**E**) RPH; (**F**) ZJH; (**G**) TTH; (**H**) EJH; (**I**) LSH; (**J**) CRH; (**K**) LMH; (**L**) COH; (**M**) Polyfloral honey.
